# T cells are blind to the dark mechanics of tumors

**DOI:** 10.1016/j.treopn.2026.05.007

**Published:** 2026-09

**Authors:** Marco Fritzsche, Karsten Kruse

**Affiliations:** 1 Kennedy Institute of Rheumatology, University of Oxford, Roosevelt Drive, Oxford OX3 7FY, UK; 2 Rosalind Franklin Institute, Harwell Campus, Didcot OX11 0FA, UK; 3 Department of Biochemistry, University of Geneva, Geneva, Switzerland; 4 Department of Theoretical Physics, University of Geneva, Geneva, Switzerland

**Keywords:** cancer, dark mechanics, force, T cell receptor, catch–slip bond

## Abstract

Tumor mechanics differ profoundly from those of healthy tissues. Yet, how abnormal mechanical properties affect T cell activation remains largely unresolved. In this opinion article, we propose that the aberrant mechanical landscape of cancer cells directly undermines early T cell activation. Substantiated by theory, we demonstrate that the lifetime of the T cell receptor–antigen bond critically depends on target cell elasticity and viscosity. By altering their viscoelastic properties, cancer cells can escape the mechanosensitive window required for T cell signaling, rendering themselves immunologically invisible. We term this phenomenon ‘dark mechanics’, a process where tumors exploit physical traits to subvert antigen discrimination, creating a mechanical route of immune evasion. This discussion suggests an opportunity to mechanically illuminate tumors for next-generation immunotherapies.

## Significance Statement

T cell surveillance depends on the mechanical context of antigen presentation. Aberrant tumor elasticity and viscosity compromise T cell receptor signaling. We introduce ‘dark mechanics’, proposing that cancer cells exploit these altered viscoelastic properties to escape the mechanosensitive window necessary for T cell activation. This mechanical cloaking provides a biophysical pathway for immune evasion, suggesting a therapeutic opportunity to mechanically illuminate tumors in next-generation immunotherapies.

## Mechanical evasion of T cell activation

T cells face a multitude of mechanical challenges as they navigate and infiltrate solid tumors 1, 2 (Box 1). The complex tumor microenvironment is structurally diverse, with mechanical properties that differ profoundly from those of healthy tissue 38, 39, 40. There is growing recognition that tumors modulate this mechanical landscape, creating spatial heterogeneity in elasticity and viscosity to actively evade the immune system 41, 42, 43, 44, 45, 46, 47, 48. Because the mechanical properties of the tumor microenvironment are fundamentally altered, one must understand how, in the context of piconewton mechanical forces generated by T cells 26, 27, 28, 29, 49, 50, 51, **T cell receptor (TCR)** (see Glossary)–**peptide major histocompatibility complex (pMHC)** signaling is affected (Box 1) and thus how activation of T cells is compromised. However, a mechanistic link detailing how these abnormal mechanics disrupt TCR antigen recognition remains poorly understood 9, 52, 53, 54.

Box 1 **Mechanics of tumor evasion**

*Mechanical barriers to T cell infiltration in solid tumors.*

The tumor microenvironment presents formidable mechanical barriers that hinder T cell infiltration 3, 4, 5. The landscape is mechanically heterogeneous, with spatial variations in cancerous tissue stiffness spanning orders of magnitude across regions 6, 7, 8. Similarly, tumor tissue fluidity and viscosity can shift dramatically compared to healthy counterparts 9, 10, 11, 12, 13, 14. T cells traversing this environment confront cancer cells that are aberrantly stiffer or softer than healthy tissue [13]. Interactions within this altered physical context may drive divergent T cell fates, sometimes contributing to exhaustion and dysfunction 15, 16, 17, while in other instances enhancing cytotoxicity 18, 19, thus complicating the immune system’s ability to clear tumors.

*Mechanical regulation of TCR–pMHC binding*

Upon confronting cancer cells, T cells continuously scan their surfaces using protrusions such as ruffles and microvilli 20, 21. During these contacts, TCR–pMHC bonds experience mechanical forces in the piconewton range 22, 23. These mechanical forces can actively stabilize the bonds via the catch-bond behavior of αβ TCRs 24, 25, a process essential for extending bond lifetimes. This force dependent kinetic proofreading amplifies the discrimination between self and nonself antigens based on bond kinetics rather than binding energies alone 26, 27, 28, 29. The ability to enhance TCR kinetics through applied mechanics is thought to be fundamental for the successful recognition of weakly binding, tumor-specific neoantigens 30, 31, 32, 33.

*Dark mechanics of solid tumors*

We propose that by altering their viscoelastic properties, cancer cells can fall outside the mechanosensitive window required for TCR triggering, effectively cloaking themselves from immune surveillance, a phenomenon we term ‘dark mechanics’. Analogous to this mechanical evasion, tumors also evade immune detection by expressing hidden, genetically encoded targets known as ‘dark antigens’ 34, 35. These antigens arise from genomic regions that are typically unexpressed in healthy cells, allowing them to escape central tolerance and remain unseen by the immune system under normal conditions 36, 37. Together, dark mechanics and dark antigens converge on a common outcome: the subversion of T cell self–nonself antigen discrimination and the impairment of T cell functions such as activation, differentiation, and cytotoxicity within tumors.

Alt-text: Box 1

In this opinion article, we extend the framework introduced before [55] to elucidate how the mechanical properties of tumors could compromise T cell activation. Specifically, we explore how aberrant spatial elasticity and viscosity of cancer cells can reduce the effective TCR–pMHC bond lifetime without altering the biochemical properties of tumor antigens. We term this phenomenon ‘dark mechanics’ (Box 1), a process where cancer cells exploit physical traits to render themselves immunologically invisible. Exploiting a theoretical description to substantiate our arguments, we find a critical dependence of T cell signaling on the magnitude, frequency, and viscoelastic anchoring of mechanical forces, leading to the discovery of a narrow **mechanosensitive window** required for the recognition of antigens by T cells. We conclude by outlining how integrating target cell mechanics deepens the understanding of immune evasion and describe implications for mechanically illuminating tumors in next-generation immunotherapies.

## Theory suggests that cancer cell mechanical properties affect early T cell activation in tumors

**Figure 1:**
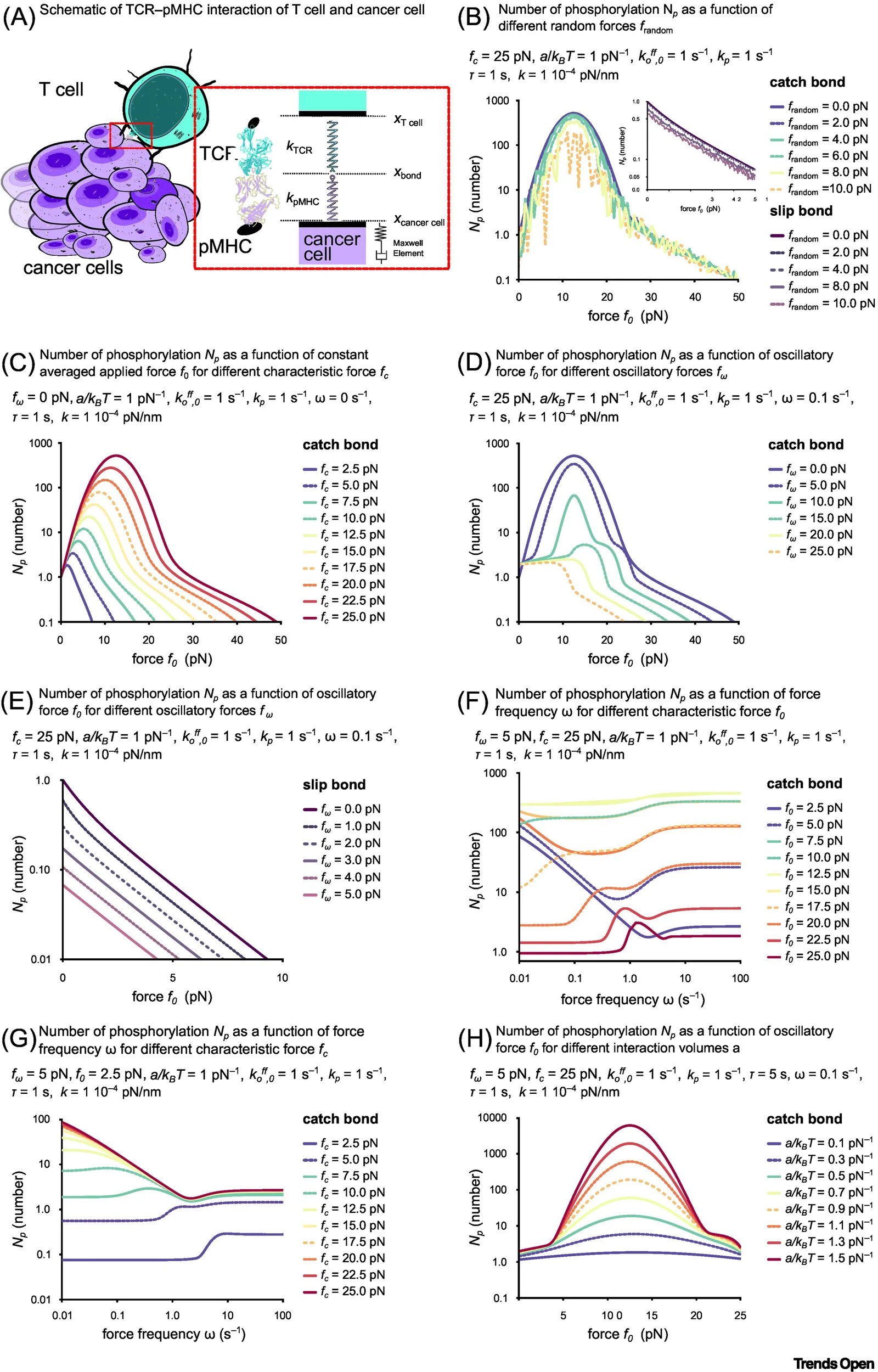
Viscoelastic coupling of pMHC to cancer cell shapes TCR–pMHC signaling. (A) Schematic of the model: a T cell interacts with a cancer cell through TCR–pMHC. Number of phosphorylation events *N_p_* for a single TCR–pMHC bond coupled to a viscoelastic cell surface. (B) *N_p_* as a function of average applied force *f_0_* with random fluctuations for a catch bond (main) and slip bond (inset). (C) *N_p_* versus static force *f_0_* for a catch bond with varying characteristic force *f^c^*. (D) *N_p_* versus average force *f_0_* under an oscillatory force with varying amplitude *fω* for a catch bond. (E) Same as (D) but for a slip bond. (F) *N_p_* versus force frequency *ω* for a catch bond with varying average force *f_0_*. (G) *N_p_* versus force frequency *ω* for a catch bond with varying characteristic force *f^c^*. (H) *N_p_* versus average force *f_0_* for a catch bond with varying interaction parameter *a/k_B_T*. pMHC: peptide MHC; TCR: T cell receptor.

To study the role of cancer cell mechanical properties in early T cell activation in tumors, we consider an effective theoretical description of a single TCR–pMHC bond (Figure 1A). Building upon our recent discussion highlighting that the lifetime of a single TCR–pMHC bond considerably depends on the T cell receptor rigidity, as well as the average magnitude and frequency of an applied oscillatory mechanical force [55], we consider in this perspective how the mechanical properties of an antigen-presenting cell affect a single TCR–pMHC interaction. We then explore the consequences of these findings in the context of the interaction between a cancer cell and a T cell within a mechanically heterogeneous tumor.

Early T cell activation depends on intact bonds between TCRs and pMHCs. We study this process through the intermediary of TCR phosphorylation, which is known from experiments *in vitro* 29, 56, 57, 58 and *in vivo* [59] to trigger T cell activation. Explicitly, we consider the number *N*_p_ of cumulative phosphorylation events of the TCR and the constant phosphorylation rate *k*_p_ [55]. The number of TCR residue phosphorylation events *n_p_(t)* up to time *t* evolves according to:

(1) $${\dot{n}}_{p}=k_{p}\cdot P$$

where the dot indicates the time derivative, and *P(t)* is the probability for an intact TCR–pMHC bond at time *t*.

We start the system at time *t = 0*, when the bond has formed and no phosphorylation has occurred, such that *P(t = 0) = 1* and *n_p_(t = 0) = 0*. Ignoring, for the moment, the possibility of TCR–pMHC rebinding after the dissolution of the bond, the time evolution of the probability *P* follows:

(2) $$\dot{P}=-{k_{off}}^{}\cdot P$$

where *k_0ff_* is the unbinding rate of the TCR–pMHC bond. The figure of merit is the final value of *n_p_* when *t → ∞*, which gives the expected number of phosphorylation events during the lifetime of a TCR–pMHC bond. The number *N_p_* denotes, from now on, the limiting value lim_t_ → ∞ *n_p_(t)*. Importantly, the rate *k_0ff_* depends on the mechanical force *f* applied to the TCR–pMHC bond and can thus vary in time. Note, we refer to mechanical force simply as ‘force’ in the following sections.

Forces have both magnitude and direction, yet the directional dependence of TCR–pMHC catch-bond behavior has not been characterized. We, therefore, neglect this aspect and adopt an effectively one-dimensional (1D) description, in which all forces act along the TCR–pMHC axis. Another aspect that deserves further investigation is the potential effects of T cell–extracellular matrix (ECM) interactions, even though we expect them to be of minor importance for the TCR–pMHC bond lifetime.

To characterize the force dependence of *k_0ff_*, we distinguish between slip and **catch bonds**. Slip bonds break faster upon the application of a mechanical force, such that the unbinding rate *k_0ff_* increases with the applied force. In contrast, catch bonds are stabilized by an applied moderate mechanical force. We follow the Bell–Evans approach to characterize the force dependence of slip and catch bonds 60, 61. Explicitly, we take:

$k_{off}={k_{off}}_{,0}\cdot \;\exp\left\{\mathrm{af}/k_{B}T\right\}$ (slip bond)

$k_{off}={k_{off}}_{,0}\cdot \;\exp\left\{\mathrm{af}\left(f/f_{c}\;-\;1\right)/k_{B}T\right\}$ (catch bond)

In the expressions, *a* is a molecular length scale, and *k_B_T* is the thermal energy. Being molecular quantities, f_c_ should be on the order of a few pN, and *a* few nanometers (nm). The thermal energy *k_B_T*, with Boltzmann constant *k_B_*, is considered at room temperature.

The state of an intact TCR–pMHC bond is captured by the position *x_bond_*, whereas the TCR is anchored to the T cell surface at position *x_Tcell_*, and the pMHC is coupled to the cancer cell at position *x_cancer cell_*. We consider these three positions, as well as the direction of the applied force, to be aligned and focus on the dynamics along the alignment direction, such that the analysis is essentially 1D. Furthermore, we approximate the mechanical response of the TCR and the pMHC by linear springs with rigidities *k_TCR_* and *k_pMHC_*, respectively, and a common rest length Δ. Dissipation during changes of *x_Tcell_* and *x_bond_* is captured by the constants ξ_Tcell_ and ξ_bond_, which have units of friction coefficients in pN · s/nm.

Together, these considerations lead to the dynamic equations:

(3) $$\xi_{Tcell}\cdot \;{\dot{x}}_{Tcell}=f_{Tcell}-k_{TCR}\;\left(x_{Tcell}-x_{bond}-\Delta \right)$$

(4) $$\xi_{bond}\cdot \;{\dot{x}}_{bond}=k_{TCR}\left(x_{Tcell}-x_{bond}-\Delta \right)-\;k_{pMHC}\left(x_{bond}-x_{cancer\;cell}-\Delta \right)$$

for the positions *x_Tcell_* and *x_bond_*. Furthermore, *f*_Tcell_ captures the force applied by the T cell on the TCR. This force is generated by T cell ruffles and microvilli, which extend toward the cancer cell. After establishing the pMHC–TCR bond, the T cell pulls on the cancer cell such that the force on the pMHC–TCR bond increases [21]. Without loss of generality, we choose Δ = 0.

Previously, we considered the case where *x_cancer cell_* = const [55]. Nevertheless, the anchoring of the pMHC to a cancer cell’s actin cortex can be much more intricate, as the actin network is itself dynamic and can even respond actively to forces transmitted by the pMHC. To integrate the mechanical properties of the cancer cell, we explicitly consider the anchoring of the pMHC on the cancer cell surface to have both a viscous and an elastic component. We assume that the coupling is elastic on short timescales and viscous on long time scales. For simplicity, we describe the response of *x_cancer cell_* in terms of a Maxwell element (Figure 1A), which has a single characteristic timescale τ separating the viscous and elastic behavior. For a force *f* applied to the anchoring point of the pMHC on the cancer cell, we thus write:

(5) $$\xi_{cancer\;cell}\cdot \;{\dot{x}}_{cancer\;cell}=f+\tau \;\dot{f}$$

The pMHC is ultimately coupled to the cancer cell’s submembranous actin cortex. The time τ is thus related to the actin cytoskeletal viscosity, measured by the friction coefficient *ξ_cancer cell_*, and the cytoskeletal elastic modulus, captured by the spring constant *k*, through *kτ = ξ_cancer cell_*. Thus, on timescales much smaller than *τ*, we have *kx_cancer cell_* = *f*, whereas in the opposite limit, for timescales much larger than *τ*, *ξ_cancer cell_* ${\overset{\cdot }{x}}_{cancer\;cell}$ = *f*. The cancer cell viscosity *η_cancer cell_* is then given by *η_cancer cell_* = *ξ_cancer cell_*/*μ*, with *μ* being a molecular length scale. The dynamic equations (3–5) will be solved for standard parameter values that are kept fixed unless noted otherwise. The values were taken from experiments whenever possible, with *a/k_B_T = 1 pN*^*-1*^ for *a = 4* nm and *k_B_T = 4 pN · nm* 56, 62, μ = 1 nm, *k_pMHC_* = *k_TCR_* = 0.1 pN/nm [55], *k = 10*^*-4*^ pN/nm, ξ_TCR_ = ξ_bond_ = 10^-4^ pN/(nm/s), *ξ_cancer cell_* = 10^-4^ pN/(nm/s), such that *τ* = 1 *s* [63], and *k_0ff, 0_ = k_p_ = 1 s*^*-1*^ 64, 65. Note that the description does not account for the mechanical properties of the T cell, which we assume to only exert the force *f*. In general, one can expect feedback from the cancer cell onto the T cell, which could influence the force in a way that depends on the T cell’s mechanical properties. Since we consider a large range of force frequencies and amplitudes, possible feedback effects are captured implicitly in the analysis.

Having introduced the description of TCR phosphorylation, we will first analyze the effects of the viscoelastic coupling to the cancer cell on the dependence of *N_p_* on various properties of the applied force and the unbinding rate *k_0ff_*. We will then consider these dependencies in the presence of TCR–pMHC rebinding, and finally, study the dependence of *N_p_* on the cancer cell’s viscoelastic mechanical properties.

### Viscoelastic anchoring of pMHC on cancer cells affects TCR–pMHC signaling

First, we considered the response of the TCR–pMHC system to an average force *f_0_* with superimposed random force fluctuations drawn uniformly from the interval (−*f*_random_, *f_random_*). In the case of a catch bond, there is an optimal average force that maximizes *N_p_*. The dependence of *N_p_* on *f_0_* is essentially independent of the amplitude of the superimposed force fluctuations (Figure 1B). This behavior stands in contrast to that of a rigid cancer cell, where fluctuations significantly alter the response [55]. As expected, for a corresponding slip bond, any applied force simply reduces *N_p_* (Figure 1B inset).

As the force fluctuations have only a minor effect on *N_p_*, we will consider the deterministic case from now on. For a catch bond and in the case of a static applied force, the value of the optimal average force and the maximal number of phosphorylation events depend on the value of the characteristic force *f_c_* (Figure 1C). Specifically, the optimal force scales linearly with *f_c_* such that *f*_0,opt_ *≈ f_c_/2*. More dramatically, the maximal value *N_p_* increases exponentially with *f_c_*.

Still, in the case of a catch bond, but now applying an oscillatory force, we find that an increasing force amplitude *f_ω_* reduces the maximum *N_p_*. For force amplitudes *f_ω_* exceeding a threshold of approximately 10 pN, the peak is supplanted by a shoulder that shifts to lower average forces *f_0_* as *f_ω_* increases further (Figure 1D). In contrast, for a slip bond under an oscillatory force, *N_p_* decreases as both the average force and the force amplitude increase (Figure 1E).

The dependence of *N_p_* on the force frequency is complex and nonmonotonic, a feature not seen with rigid cells [55]. As the average force *f_0_* increases, the dependence of *N_p_* on frequency shifts from exhibiting a pronounced minimum around 1 s^-1^ to a local maximum at that same frequency (Figure 1F). The maximal value of *N_p_* is achieved for intermediate values of approximately 12 pN. As a function of the characteristic force *f_c_*, the number of phosphorylation events increases with *f_c_* (Figure 1G). Finally, the interaction volume parameter *a/k_B_T* also influences the phosphorylation number; a shoulder develops in the dependence of *N_p_* on *f_0_* for values of *a/k_B_T* greater than approximately 0.7 pN^-1^, which is similar to the effect seen with increasing force amplitude (Figure 1H).

In conclusion, the viscoelastic nature of the coupling of the pMHC on the cancer cell surface creates an intricate signaling response that depends on the interplay between the average force, amplitude, and frequency of the forces applied by the T cell. This complex behavior is a direct consequence of the cell’s mechanical properties and stands in contrast to the simpler force response of an idealized rigid cell [55].

### Rebinding of TCR to pMHC improves TCR–pMHC signaling

**Figure 2:**
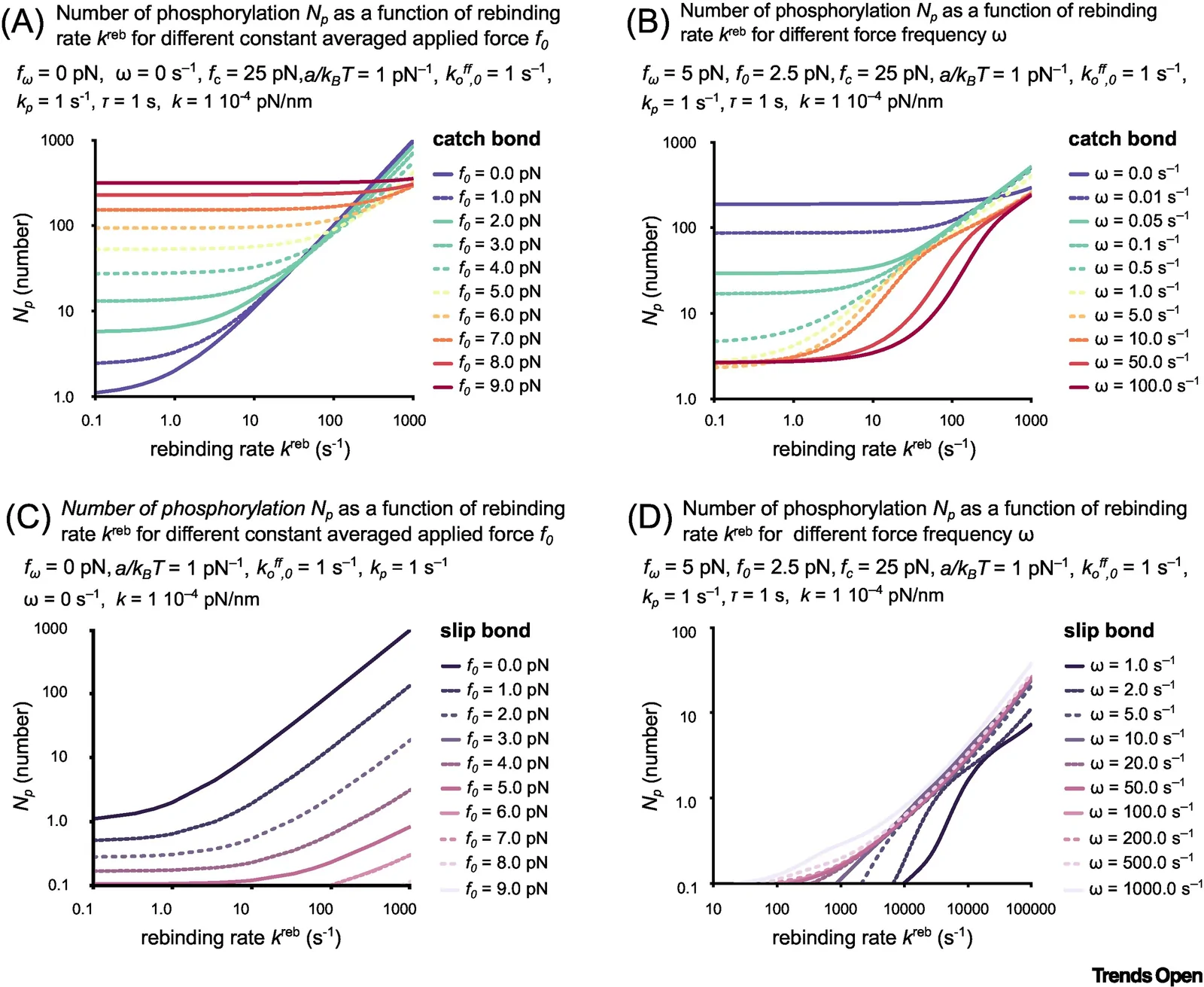
Rebinding enhances TCR–pMHC signaling under force. Number of phosphorylation events *N_p_* as a function of the rebinding rate *k_reb_* or force frequency *ω*. (A) *N_p_* versus *k_reb_* for a catch bond under a static force for different characteristic forces *f_c_*. (B) *N_p_* versus *k_reb_* for a catch bond under an oscillatory force for different force frequencies *ω*. (C) *N_p_* versus *ω* for a slip bond with different average applied forces *f_0_*. (D) *N_p_* versus *k_reb_* for a slip bond under a static force for different average applied forces *f_0_*.

When a TCR remains near a target pMHC after bond dissociation, for instance, if receptor–ligand diffusion is slow compared to reaction kinetics, TCR–pMHC rebinding can occur. In such a case, one expects the number of phosphorylation events *N_p_* to increase with the rebinding rate. This is indeed the case (Figure 2). Nevertheless, compared to the case of rigid anchoring [55], viscoelastic anchoring of the pMHC to a cancer cell surface changes the dependence of *N_p_* on parameters in a subtle way.

For catch bonds, the dependence of *f_0_* is nonmonotonic for some values of *k_reb_* (Figure 2A). In contrast, under an oscillatory force, *N_p_* decreases monotonically as the force frequency ω increases, except for very large values of *k_reb_* (Figure 2B). For slip bonds subjected to a constant force *f_0_, N_p_* also increases with the rebinding rate *k_reb_* (Figure 2C). For oscillatory forces and extremely large frequencies, *N_p_* can increase with increasing force frequency (Figure 2D). Such very large rebinding rates might become relevant when considering an immunological synapse with 10–100 TCRs per cluster during full T cell activation.

### Effects of tumor mechanics on TCR–pMHC signaling

Beyond the effects of the viscoelastic molecular anchoring of the pMHC to the cancer cell, we now explore the influence of the phenomenological mechanical properties of the cancer cells themselves. We show that cancer cells might become immunologically invisible or ‘dark’ by shifting their mechanical properties away from the range of healthy tissue 66, 67 and outside the optimal range for early T cell activation.

**Figure 3:**
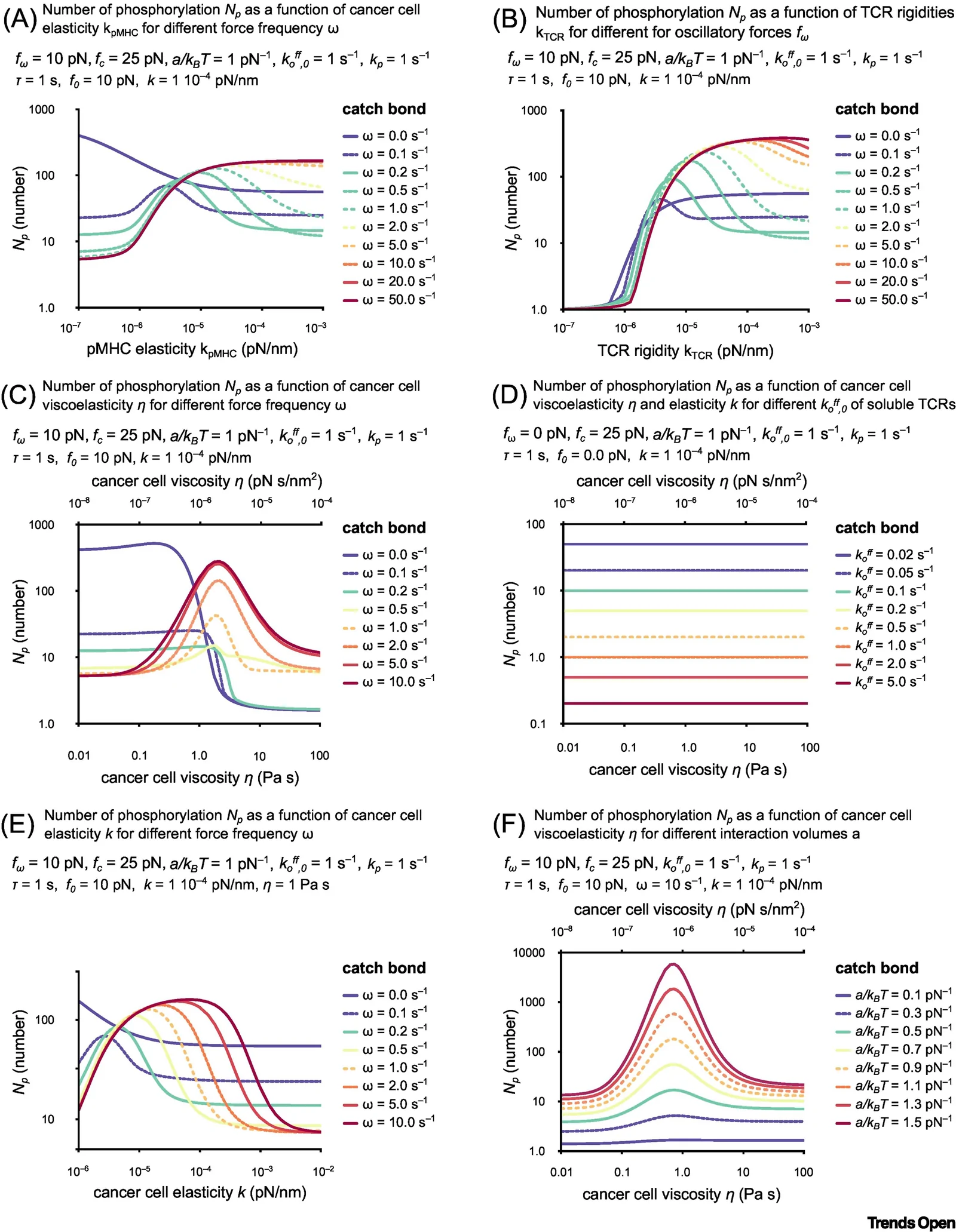
Tumor mechanics modulate TCR–pMHC signaling. (A) *N_p_* as a function of pMHC rigidity *k*_pMHC_ for different force frequencies *ω*. (B) *N_p_* as a function of TCR rigidity *k_TCR_* for different force frequencies *ω*. (C) *N_p_* versus cancer cell viscosity *ξ_cancer cell_* for different force frequencies *ω*. (D) For soluble TCRs, *N_p_* as a function of cancer cell viscosity for different unbinding rates *k_off__,0_*. (E) *N_p_* as a function of cancer cell elasticity *k* for different force frequencies *ω*. (F) *N_p_* versus cancer cell viscosity for different interaction parameters *a/k_B_T*. TCR: T cell receptor.

First, we examined the influence of pMHC rigidity *k_pMHC_* on TCR phosphorylation. The number of phosphorylation events, *N_p_*, is largely insensitive to this parameter as long as the rigidity remains above approximately 1 × 10^-4^ pN/nm (Figure 3A). Below this rigidity, nevertheless, signaling collapses for sufficiently fast oscillating applied forces, with *N_p_* dropping by over an order of magnitude. A similar insensitivity is observed for TCR rigidity *k_TCR_* above approximately 1 × 10^-4^ pN/nm (Figure 3B), in contrast to the rigid cell [55]. Similarly to the pMHC, signaling fails for softer TCRs. Changing the viscosity of the cancer cell reveals a much more dramatic effect on the number of phosphorylation events than changes in its rigidity.

Above a force frequency of *ω* = 0.1 s^-1^, *N_p_* displays a sharp peak at an optimal cancer cell viscosity (*η_cancer cell_* ≈ 1–10 Pa · s), where signaling is enhanced by several orders of magnitude (Figure 3C). Interestingly, healthy tissue cells have a typical bulk viscosity of approximately 0.1–5 Pa · s, while cancer cells have a typical bulk viscosity of approximately 1–100 Pa · s 13, 66, 68, 69. Deviating from this optimal viscosity causes *N_p_* to plummet, immunologically cloaking the tumor and turning it dark. For lower force frequencies, TCR phosphorylation is restricted to an even smaller viscosity range. Crucially, this viscosity window is entirely force-dependent; in the absence of applied force, as with soluble TCRs, *N_p_* is completely independent of both the cell’s viscosity and its elasticity (Figure 3D). Consistent with cancer cell viscosity, changing the cancer cell elasticity reveals a similar window (Figure 3E). One could speculate that cancer cells actively modulate their mechanical properties to evade T cell recognition, for example, by changing the activation of motor proteins or crosslinkers. Finally, the sensitivity of this process is also modulated by the bond’s intrinsic molecular properties, as the sharpness and height of the signaling peak are strongly dependent on the interaction volume of the TCR–pMHC bond with parameter *a/k_B_T* (Figure 3F).

## Implications of abnormal mechanical properties of cancer cells for early T cell activation in tumors

The analysis indicates that tumor mechanics play a crucial role in early T cell activation: there is a narrow window in which cancer cell viscosity and the frequency of applied forces by T cells to the TCR–pMHC bond between the T cell and cancer cell must be matched for robust signaling. Mismatches effectively cloak antigens (Figure 3). TCR–pMHC rebinding can amplify signaling but remains constrained by the same mechanical context (Figure 2). The mechanical heterogeneity of tumors, which mix stiff and soft cancer cells, may avoid the emergence of specific ‘anticancer T cells’ and thus poses an important challenge to the design of cancer immunotherapy.

## Potential limitations of the theoretical analysis

This discussion provides a conceptual framework for studying the effects of mechanical properties on early T cell activation and suggests the existence of mechanically dark tumors. Nevertheless, it is built upon several simplifications that represent limitations. First, our analysis is restricted to a single TCR–pMHC bond, thereby neglecting the cooperative effects and complex signaling integration that occur within the broader immunological synapse, which involves TCR clustering and coreceptor engagement 29, 70. Second, we describe the cancer cell’s mechanical response using a simple Maxwell element. The true mechanical behavior of cells can be more complicated, as it depends on the mechanical interactions between many different cell components, including the cytoskeleton, the plasma membrane, and the cytosol. Consequently, the cellular mechanical response is typically nonlinear and involves more than one timescale. Similarly, the T cell’s mechanical contributions are summed up in a prescribed externally applied force, thus neglecting the dynamic mechanical response of the T cell’s cytoskeleton and protrusions during target scanning [21]. Experimental data are currently insufficient to yield a comprehensive view of the cells’ mechanical features. Given the generic aspects captured by our approach, though, we anticipate that our main conclusions will remain valid.

Third, the phosphorylation rate, *k_p_*, is assumed to be constant, whereas it could itself be force-dependent or subject to regulatory feedback. In particular, this approach represents a strong simplification of the full kinetic proofreading cascade [27]. Finally, the presented 1D analysis considers only forces applied along the bond axis, omitting the complex three-dimensional (3D) forces that exist in a 3D cellular interaction, and does not account for the mechanical pressures of surrounding cells imposed by the dense tumor microenvironment and altered ECM characteristic of solid tumors [46]. Extending the framework to multielement viscoelastic models with spatial heterogeneity and multiaxial force components will help test the robustness of the mechanosensitive window.

## Future experiments on early T cell activation in tumors

The discussion calls for new experimental approaches to directly test the mechanosensitivity of T cell activation at the single-molecule level. Advanced biophysical techniques, such as atomic force microscopy or optical tweezers combined with live-cell imaging, could be used to measure TCR–pMHC bond dynamics and subsequent signaling events on cancer cells whose mechanical properties are systematically varied using cytoskeleton-targeting drugs or micropatterned substrates. Furthermore, the development of Förster resonance energy transfer (FRET) biosensors for TCR phosphorylation could allow for real-time correlation of signaling with force application, directly testing the existence of a permissive window of mechanical T cell activation (Figure 3B). Such experiments would be crucial to validate the link between target cell mechanics and the very first steps of antigen recognition 71, 72, 73.

As a concrete roadmap, future experiments could independently vary cancer cell viscosity, pharmacologically or genetically targeting the cytoskeleton, and applied force frequency through actomyosin tuning or cyclic substrate actuation, while applying calibrated piconewton loads with optical traps or atomic force microscopy, and reading out single TCR phosphorylation using FRET reporters. Mechanically tunable tumor organoids, biopsies, or intravital force reporter approaches could then assess whether the mechanosensitive window persists in tissue contexts and during therapy response.

## Mechanically illuminating tumors for immunotherapy

The findings of the perspective motivate mechanical tumor profiling as a companion diagnostic to identify mechanically nonpermissive tumors that suppress robust T cell activation. Recognizing the importance of dark mechanics also opens several avenues for developing therapeutic strategies to improve cancer immunotherapy. Concretely, this can be achieved by utilizing (i) reagents that normalize tumor mechanics to reopen the mechanosensitive window and (ii) high-affinity, affinity-driven soluble TCR or antibody agent platforms that decouple antigen engagement from T cell mechanosensation, thereby overcome a primary activation barrier in mechanically suppressive tumor settings.

At the outset, restoring the immunological visibility of tumors is a key therapeutic goal. For strategy (i), one plausible approach could involve the development of ‘mechanotherapeutics’ [74], agents designed to normalize the viscoelastic properties of cancer cells, thereby reopening the mechanosensitive window for T cell recognition. Such mechano-modulating drugs could act synergistically with existing treatments, rendering previously resistant tumors susceptible to checkpoint inhibitors or CAR T cell therapies. An alternative strategy is to engineer the T cells themselves. Future generations of CAR T cells could be designed with customized force-generation profiles or activation machinery tuned to the specific mechanical landscape of a patient’s tumor.

For strategy (ii), soluble reagents are mechanically nonpermissive. Since a dense ECM can limit macromolecule penetration, combining soluble TCRs with ECM-modulating strategies could further improve intratumoral access. An additional option beyond modifying the mechanical properties of tumors or decoupling T cell activation from mechanics would be to engineer TCRs or CARs 75, 76 and their membrane anchoring to broaden the mechanosensitive window of T cell activation and thus restore immunological visibility.

## Concluding remarks

We emphasize again that the process of self–nonself antigen recognition between a T cell and a cancer cell should be viewed not just as a biochemical event, but as a dynamic mechanical process that occurs at a mechanoimmunological synapse in the context of a constantly evolving mechanical tissue landscape. The mechanical properties of healthy tissues are tightly regulated, and this mechanical homeostasis may be essential for proper immune surveillance. The mechanical properties of tumors have evolved to evade this surveillance. By integrating mechanical context into models of T cell antigen discrimination, we aim to make the immuno-oncology field aware of the functional significance of mechanical force for T cell self–nonself antigen recognition in solid tumors and identify new mechanobiological principles that could inform the next generation of immunotherapies (see Outstanding questions). This analysis reveals the existence of a mechanosensitive window in a generic setting. The window’s precise properties will depend on the details of the investigated biological system. Further experiments will have to demonstrate whether cancer cells indeed exploit this strategy to render themselves immunologically invisible. Illuminating the ‘dark mechanics’ will be essential to map the full extent of tumor immune evasion, providing a rational biomechanical basis for the design of future immunotherapies.

Outstanding questions How do specific mechanical properties, such as the distinct contributions of viscosity and elasticity, differentially impact T cell receptor triggering and downstream signaling cascades?

Do different tumor types exhibit distinct ‘mechanical signatures’ of viscoelasticity that correlate with their immunogenicity and response to checkpoint blockade?

Can pharmacological targeting of the tumor cytoskeleton be used to normalize cancer cell mechanics and restore T cell recognition *in vivo*?

How does the ‘dark mechanics’ phenomenon integrate with other known immune evasion mechanisms, such as checkpoint inhibition and antigen loss?

Does the mechanosensitive window for activation vary between different T cell subsets (e.g., CD4+ vs. CD8+) or differentiation states?

Alt-text: Outstanding questions

Key references

1. Fuhs, T. *et al*. (2022) Rigid tumors contain soft cancer cells. *Nat. Phys.* 18, 1510–1519.

Shows that bulk tumor stiffness does not reflect individual cancer cell mechanics; viscoelasticity is the key parameter cancer cells may exploit to evade T cell recognition.

2. Feng, Y. *et al*. (2017) Mechanosensing drives acuity of αβ T cell recognition. *Proc. Natl. Acad. Sci. U.S.A.* 114, E8204–E8213.

Demonstrates that mechanical force is essential for TCR antigen discrimination, motivating the ‘dark mechanics’ concept.

3. Das, D.K. *et al*. (2015) Force-dependent transition in the T cell receptor β-subunit allosterically regulates peptide discrimination and pMHC bond lifetime. *Proc. Natl. Acad. Sci. U.S.A.* 112, 1517–1522.

Provides the molecular basis for TCR catch-bond behavior: piconewton forces extend bond lifetime via conformational change, which is key to understanding how cancer cell viscoelasticity impairs signaling.

4. Fritzsche, M. and Kruse, K. (2024) Mechanical force matters in early T cell activation. *Proc. Natl. Acad. Sci. U.S.A.* 121, e2404748121.

Establishes that the TCR–pMHC bond lifetime is acutely sensitive to the magnitude and frequency of force; the present work extends this framework to the tumor context.

5. Reinherz, E.L. *et al*. (2023) Harnessing αβ T cell receptor mechanobiology to achieve the promise of immuno-oncology. *Proc. Natl. Acad. Sci.* 120, e2215694120.

Articulates the translational case for TCR mechanobiology in cancer immunotherapy, supporting the claim that countering ‘dark mechanics’ could inform next-generation therapies.

Alt-text: Key references

## Author Contributions

M.F. and K.K. jointly developed the conceptualization for this opinion article. M.F. and K.K. contributed equally to writing the original draft. Both authors contributed to the critical review and editing of the final version. All authors have read and approved the final manuscript.

## Glossary

**Catch bonds**: noncovalent receptor–ligand interactions whose lifetimes initially increase under applied mechanical tensile force, are essential for stabilizing T cell receptor binding to specific antigens.
**Mechanosensitive window**: a specific, narrow range of physical tissue properties (such as stiffness and viscosity) within which T cell receptor signaling and effective antigen recognition can optimally occur.
**Peptide MHC (pMHC**): a molecular complex displayed on the surface of target cells, consisting of a peptide fragment bound to a major histocompatibility complex protein, serving as the specific target for T cell receptors.
**T cell receptor (TCR**): a complex of integral membrane proteins on the surface of T cells, responsible for recognizing specific antigen fragments presented by MHC molecules and initiating the immune activation cascade.
**Viscoelasticity**: a material property encompassing both fluidlike (viscous) and solidlike (elastic) characteristics during deformation. In biological tissues, it governs how cells resist, transmit, and respond to mechanical forces.
